# How Transcranial Direct Current Stimulation Can Modulate Implicit Motor Sequence Learning and Consolidation: A Brief Review

**DOI:** 10.3389/fnhum.2016.00026

**Published:** 2016-02-10

**Authors:** Branislav Savic, Beat Meier

**Affiliations:** ^1^Institute of Psychology, University of BernBern, Switzerland; ^2^Center for Cognition, Learning, and Memory, University of BernBern, Switzerland

**Keywords:** non-invasive brain stimulation, transcranial direct current stimulation, serial reaction time task, implicit motor sequence learning, memory consolidation

## Abstract

The purpose of this review is to investigate how transcranial direct current stimulation (tDCS) can modulate implicit motor sequence learning and consolidation. So far, most of the studies have focused on the modulating effect of tDCS for explicit motor learning. Here, we focus explicitly on implicit motor sequence learning and consolidation in order to improve our understanding about the potential of tDCS to affect this kind of unconscious learning. Specifically, we concentrate on studies with the serial reaction time task (SRTT), the classical paradigm for measuring implicit motor sequence learning. The influence of tDCS has been investigated for the primary motor cortex, the premotor cortex, the prefrontal cortex, and the cerebellum. The results indicate that tDCS above the primary motor cortex gives raise to the most consistent modulating effects for both implicit motor sequence learning and consolidation.

Many of our everyday activities are organized into sequences, some deliberate, some simply by coincidence. Getting up and ready for work, writing a scientific paper, or doing leisure activities often follow repeated sequences of events. Many of these sequences are established incidentally rather than intentionally, that is, learning is implicit (Cleeremans et al., 1998). The implicit acquisition of sequences often involves a motor component and thus, it is termed implicit motor sequence learning (but see Meier and Cock, 2010; Weiermann et al., 2010 for non-motor implicit sequence learning tasks). After acquisition, performance can become resistant to decay, that is consolidated. In recent years, transcranial direct current stimulation (tDCS) has been used to enhance performance in a variety of learning and memory tasks in healthy participants, but the majority of the studies focused on explicit rather than implicit sequence learning tasks and on learning rather than consolidation (Coffman et al., 2014; Shin et al., 2015). Therefore, there is no clear consensus on how tDCS enhances implicit motor sequence learning and consolidation. The aim of this article is to review the evidence for modulating effects of tDCS on implicit motor sequence learning and consolidation.

## Implicit motor sequence learning and consolidation

Implicit motor sequence learning is typically tested with the serial reaction time task (SRTT), originally introduced by Nissen and Bullemer (1987). In this paradigm, a sequence of correct response key presses follows the sequence of designated target locations. Unbeknownst to participants, the order of target locations follows a sequence predetermined by the experimenter. With practice, performance gets faster compared to a randomized control condition. If the sequence is switched to random, performance is slowed again. These changes are taken as evidence of implicit sequence learning.

Typically, two kinds of learning are involved, general motor skill (GMS) learning and sequence-specific (SS) learning (Meier and Cock, 2014; cf. Janacsek and Nemeth, 2013). GMS learning refers to the acquisition of expertise with the general requirements of the task^1^. It can be measured as the speed up of reaction times (RT) across blocks, see Figure 1①. SS learning can be measured as the RT difference between a random block that occurs after a sequence has been presented several times and the surrounding sequenced blocks. This disruption score is an indirect measure of SS learning, see Figure 1②.

**Figure 1:**
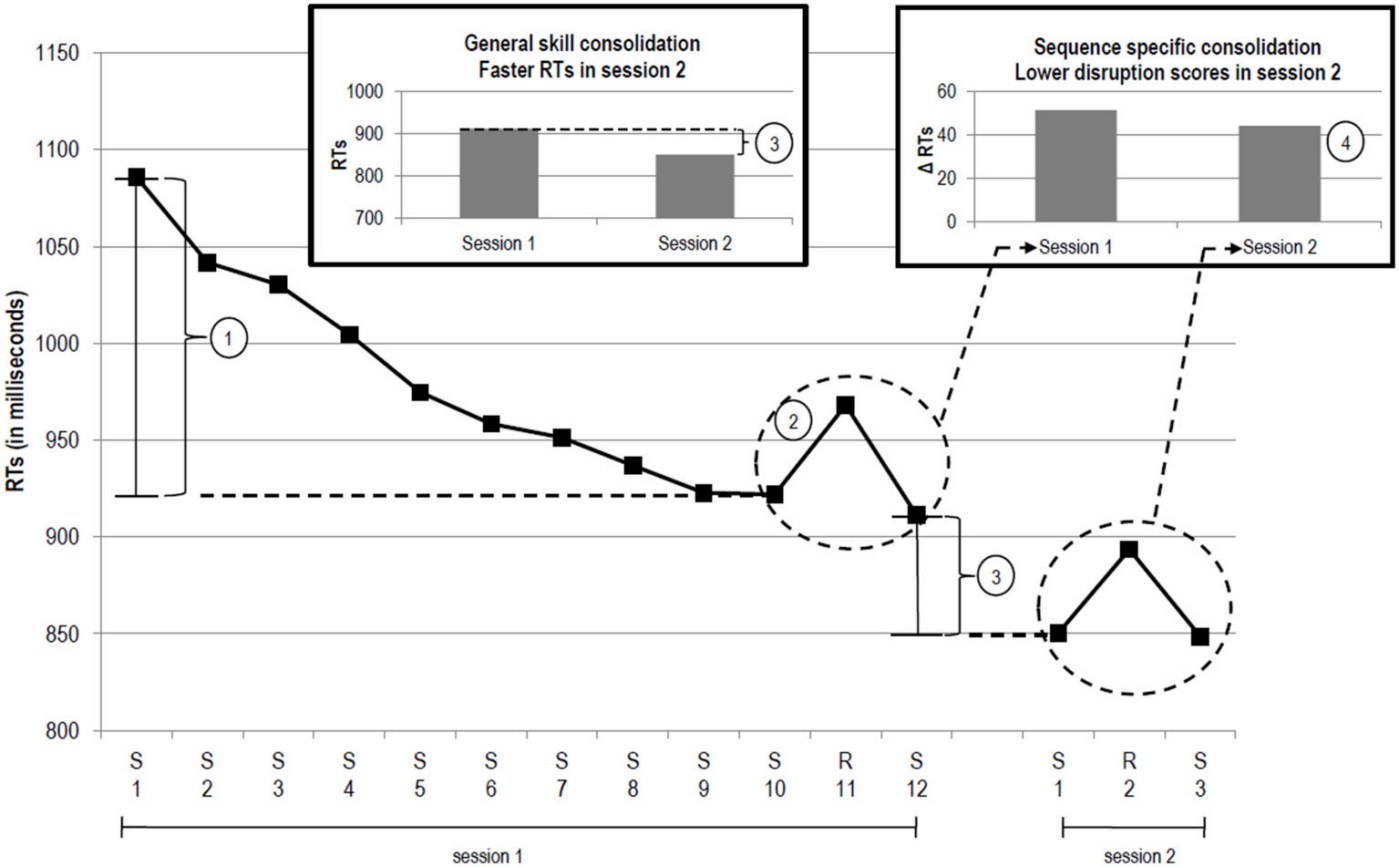
**Prototypical performance trajectory in the SRTT (adapted from Meier and Cock, 2014)**. The x-axis depicts RTs across blocks (“S” sequenced block, “R” random block). General motor skill learning (RT difference between S 1 and S 10). Sequence-specific learning (i.e., disruption score calculated as RT difference between R 11 and the mean of S 10 and S 12). General motor skill consolidation (RT difference between S 12 of session 1 and S 1 of session 2). Sequence-specific consolidation (RT difference between the disruption scores of the two sessions).

With time passing and without further practice performance can become robust, resistant to decay and interference, that is, consolidated (Shadmehr and Brashers-Krug, 1997; Krakauer and Shadmehr, 2006). Memory consolidation can be conceptualized as performance improvement or maintenance across sessions (Robertson et al., 2004). Consolidation can be assessed by repeating the SRTT in a second session separated by a period of time in which participants are not engaged with the SRTT. GMS consolidation can be measured as the mean RT difference between the last sequenced block of session one and the first sequenced block of session two, see Figure 1③. SS consolidation can be measured as the mean difference between the disruption scores of the two sessions, as depicted in Figure 1④ (for reviews on consolidation see Doyon et al., 2009; Robertson, 2009; Siengsukon and Boyd, 2009; Song, 2009; Dayan and Cohen, 2011).

### Transcranial direct current stimulation (tDCS)

Through the application of a current between two electrodes (i.e., an anode and a cathode) tDCS can modulate cortical excitation (Nitsche and Paulus, 2000, 2001). Typically, anodal tDCS is thought to induce subthreshold membrane depolarization, and cathodal tDCS is thought to induce hyperpolarization, respectively (Nitsche et al., 2003a; Bikson et al., 2004; Ruffini et al., 2013). Moreover, it has been suggested that tDCS modulates mechanisms of cortical plasticity, which in turn modify the synaptic bonds between neurons (Fritsch et al., 2010; Stagg et al., 2011). As tDCS modulates cortical plasticity and cortical plasticity is generally involved in learning, the application of tDCS may have the potential to enhance or diminish learning (Rioult-Pedotti et al., 2000; Liebetanz et al., 2002). Particularly, anodal tDCS is thought to enhance learning and cathodal tDCS is thought to diminish it. The immediate effect of tDCS can outlast stimulation for more than 1 h dependent on parameters such as current strength, stimulation duration, electrode size, and inter-electrode distance (Shin et al., 2015). Reducing the electrode size increases the spatial resolution of stimulation, in other words a smaller electrode modulates a smaller area of the cortex underneath it (Nitsche et al., 2007; Bastani and Jaberzadeh, 2013).

The active electrode is placed on the scalp above the cortical area that is to be modulated with tDCS and the return electrode is placed above the contralateral side either on inactive or active regions. Inactive regions should not modulate cortical areas, for example the shoulder, while active regions should modulate cortical areas, for example the motor cortex. Placing the return electrode on an inactive region reflects a unilateral setting (i.e., only one hemisphere is stimulated). In contrast, placing the return electrode on an active region reflects a bilateral setting (i.e., both hemispheres are stimulated, see Nasseri et al., 2015 for an overview of tDCS settings). Importantly, for motor cortex stimulation the active electrode is usually placed above the motor cortex and the return electrode above the eyebrow (i.e., supraorbital region). This setting is considered bilateral because human head model studies show that the return electrode placed above the supraorbital region modulates cortical areas (Miranda et al., 2006; Laakso et al., 2015). In addition, two kinds of application can be distinguished. tDCS applied during the execution of a particular task (e.g., the SRTT) is termed on-line stimulation, tDCS applied before the execution of a particular task (e.g., the SRTT) is termed off-line stimulation. As a control condition, typically sham stimulation is used, during which current is delivered only for 30 s which has no effect on the neural population. Importantly, from a subjects' point of view, sham cannot be distinguished from real stimulation (Gandiga et al., 2006).

## Methods

We focus on studies in which implicit motor sequence learning and/or consolidation were addressed with the SRTT. The use of the SRTT was the critical criterion because the SRTT is the classic task for implicit motor sequence learning which gives reliable results that have been replicated consistently. In order to select the relevant studies, PubMed was used as a search engine, with “tDCS” and “implicit motor sequence learning,” “tDCS” and “consolidation,” and “tDCS” and “SRTT” as keywords. A total of six studies conformed to the search criteria. Five of them tested the influence of tDCS on frontal brain areas (in particular the motor and premotor cortex) and one of them tackled the cerebellum. Table 1 shows an overview of these studies. Moreover, in order to make a comparison across studies possible, the critical learning and consolidation effects in milliseconds are also provided.

**Table 1:** **Summary of the studies aiming to modulate implicit motor sequence learning and memory consolidation with tDCS**.

**Author**	**Hand tested**	**Sequence length**	**Sequence repetition (total number of trials per sequenced blocks)**	**Calculation**	**tDCS application**	***N* (per condition)**	**Stimulation parameters**	**Electrodes size**	**Manipulation of electrode setting**	**Electrode setting**		**Effects in milliseconds (ms)**				**Significance**			
												**Learning**		**Consolidation**		**Learning**		**Consolidation**	
										**Anode**	**Cathode**	**GMS**	**SS**	**GMS**	**SS**	**GMS**	**SS**	**GMS**	**SS**
Nitsche et al., 2003b	Right	12 items	720	RTs of each block were divided by the RTs of block one	On-line	20	1 mA; 15 min	35 cm^2^	Between; stimulation type (anodal, cathodal, sham) manipulate within	Left M1	Right supraorbital region	(10) 15	(40) 60	**n.m**.	**n.m**.	↑	↑	**n.m**.	**n.m**.
										Left PM	Right supraorbital region	(25) 0	(55) 60			✘	✘		
										Left lateral PFC	Right M1	(10) 5	(50) 60			✘	✘		
										Left medial PFC	Right M1	(0) 0	(40) 50			✘	✘		
										Right supraorbital region	Left M1	(10) 5	(40) 60			✘	✘		
										Right supraorbital region	Left PM	(25) 10	(55) 50			✘	✘		
										Right M1	Left lateral PFC	(10) 5	(50) 50			✘	✘		
										Right M1	Left medial PFC	(0) 0	(40) 40			✘	✘		
Kuo et al., 2008	Right	12 items	720	“Standard” defined as in Figure 1	Off-line	12	1 mA; 10 min	35 cm^2^	Between; stimulation type (anodal, cathodal, sham) manipulate within	Left M1	Right supraorbital region	(10) 10	(55) 50	**n.m**.	**n.m**.	✘	✘	**n.m**.	**n.m**.
										Right supraorbital region	Left M1	(20) 10	(45) 35			✘	✘		
Kang and Paik, 2011	Right	12 items	1200	Disruption scores measured as ratio between RTs in sequenced and random blocks	On-line	11	2 mA; 20 min	25 cm^2^	Within	Left M1	Right supraorbital region	(80) 10	(60) 70	(−30) 5	(30) −15	**n.m**.	✘	**n.m**.	↑
										Left M1	Right M1	(80) 15	(60) 85	(−30) 10	(30) 10		✘		↑
Kantak et al., 2012	Left	10 items	600	Disruption scores measured as ratio between RTs in sequenced and RTs random blocks	On-line	13	1 mA; 15 min	8 cm^2^(Anode)	Within	Right M1	Left supraorbital region	(60) 70	(100) 160	(−80) 10	(10) 20	↑	✘	**n.m**.	**(↑)**
								48 cm^2^ (Cathode)		Right dorsal PM	Left supraorbital region	(60) 60	(100) 140	(−80) −50	(10) 20	✘	✘		✘
Nitsche et al., 2010^*^	Right	12 items	720	RTs of each block were divided by the RTs of block one	Off-line	20(Experiment 1) 12(Experiment 2) 32(Experiment 3)	1 mA; 15 min	35 cm^2^	None	Left PM	Right supraorbital region	(20) 0	(55) 55	(−50) 0	(45) −20	✘	**n.m**.	↑	**n.m**.
Ferrucci et al., 2013	Both hands	12 items	264	“Standard” defined as in Figure 1	Off-line	21	2 mA; 20 min	35 cm^2^	None	Cerebellum (2 cm below inion)	Right arm	(200) 190	(25) 35	(−10) 150	(5) −5	**n.m**.	**n.m**.	↑	↑

*GMS, general motor skill; SS, sequence-specific. The effects in ms are measured as depicted in Figure 1, and they were extracted from graphs. For each effect in ms in brackets the ms for the sham tDCS groups, and outside the bracket the ms for the actual tDCS groups. If the method of calculation is different from Figure 1 the calculation is described in the column “Calculation.” ↑, tDCS enhanced the respective SRTT component; ✘, no influence; n.m., not measured*.

* *Because of the design complexity of this study, in Table 1 only the tDCS condition that was significantly different from sham is described*.

## Results

In a first study, Nitsche et al. (2003b) investigated whether *on-line* tDCS modulates implicit sequence *learning*. tDCS was applied to one of four brain areas of the left hemisphere, the motor cortex (M1), the premotor cortex (PM), the lateral, and the medial prefrontal cortex (PFC). Specifically, for M1 and PM stimulation the return electrode was placed above the right supraorbital region, and for both PFC stimulations it was placed above the right M1. The results showed that anodal tDCS above the M1 enhanced GMS learning, indicated by faster RTs in the sequenced blocks compared to sham. Furthermore, anodal tDCS above the M1 enhanced SS learning, indicated by a bigger RT difference between random and surrounding sequenced blocks compared to sham. tDCS above the other areas did not affect learning at all.

Kuo et al. (2008) investigated whether *off-line* tDCS also modulates *learning* with either anodal or cathodal stimulation. A further aim was to evaluate pharmaceutical interventions, however, here we focus on the placebo conditions. For the anodal montage, the active electrode was placed above the left M1 and the return electrode was placed above the right supraorbital region. For the cathodal montage the reverse setup was used. tDCS started and ended before the SRTT. The results showed that neither anodal nor cathodal tDCS affected SRTT performance. Thus, offline tDCS over M1 did not modulate sequence learning at all.

Kang and Paik (2011) investigated the influence of two *bilateral on-line* tDCS settings above the M1 on *learning and consolidation*. For the first setting, the anode was placed above the left M1 and the cathode was placed above the right supraorbital region. For the second setting, the anode was placed above the left M1 and the cathode was placed above the right M1. In a first session, stimulation started after three blocks, continued for 11 blocks, and ended before the last three blocks. The first and the last three blocks were composed of two random and one sequenced block which were used to calculate learning. After 24 h, another three blocks were used to test consolidation. Learning and consolidation was calculated as ratio between the RTs in the sequenced and the random blocks in session one and two, respectively. The results showed that at the end of session one, the ratio decreased for all conditions, indicating similar SS learning for all conditions. In session two, the ratio was maintained in the two tDCS conditions but not in the sham condition. These results suggest that tDCS enhanced SS consolidation. However, when SRTT components for session one were calculated as RT differences between random and sequenced blocks, the disruption score for tDCS conditions was already higher initially. This makes the interpretation of a specific SS advantage for the stimulation conditions somewhat equivocal.

Kantak et al. (2012) investigated the influence of *on-line* tDCS above the M1 and above the dorsal PM cortex on *learning and consolidation*. The anode was placed above the M1 or the dorsal PM of the right hemisphere. In both groups the cathode was placed above the left supraorbital region. In a first session, tDCS started after two blocks, continued for six further blocks, and stopped before the last two blocks. The first and the last two blocks were composed of a random and a sequenced block and were used to calculate learning. After 24 h, another two blocks, one sequenced, and one random were used to calculate consolidation. The results showed that the decrease in RTs across the sequenced blocks was greater when M1 was stimulated compared to sham, indicating that anodal tDCS of M1 enhanced GMS learning. At the end of session one, the SS learning in the PM and M1 tDCS conditions was not statistically different from sham, even though there was a trend. To test consolidation, the ratio between RTs in sequenced and random blocks at the end of session one was compared to the according ratio after 24 h. This ratio was maintained in the M1 and sham groups but not in the PM group. Furthermore, in session two, the M1 group had a smaller ratio compared to PM and sham groups. Because the M1 and PM groups had already smaller ratios than the sham group at the end of session one, tDCS above the M1 may have enhanced GMS and SS learning initially and this was retained after 24 h.

Nitsche et al. (2010) investigated whether *off-line* tDCS above the PM cortex applied during sleep following learning could enhance *consolidation*. The active electrode was placed above the left PM and the return electrode was placed above the right supraorbital region. The study consisted of three experiments. In Experiment 1, two groups performed the SRTT and then went to sleep. One group was woken up during the night and was re-tested. The other group was re-tested the next morning. In Experiment 2, tDCS was delivered during an SRTT-like task that was composed of random blocks only. In Experiment 3, the same setting was used as in Experiment 1, but without sleep. In each experiment, the re-test consisted of three blocks, one random block followed by two sequenced blocks, which were used to assess consolidation. In Experiment 1, the results showed that anodal tDCS during sleep enhanced GMS consolidation, as indicated by smaller RTs in the sequenced blocks of the re-test compared to sham, but only when participants were re-tested during the night. When participants were re-tested the next morning there was no difference between the real tDCS and the sham conditions. In Experiment 2, tDCS had no effects on performance, indicating that tDCS did not influence GMS learning. In Experiment 3, tDCS had no effect on GMS learning, no effect on SS learning, and no effect on consolidation. Thus, this study provides further evidence that PM tDCS does not modulate implicit sequence learning or consolidation.

Finally, Ferrucci et al. (2013) investigated whether *off-line* tDCS of the cerebellum would enhance *consolidation*. The anode was placed above the cerebellum and the cathode was placed above the right arm. The results showed faster RTs for the tDCS group in the sequenced blocks post stimulation compared to pre stimulation. In contrast, for the sham group there was no difference. This indicates that tDCS enhanced GMS consolidation. Furthermore, post stimulation the disruption score was larger for the tDCS than for the sham group. This indicates that tDCS also enhanced SS consolidation.

## Discussion

Applying tDCS above the cortex of healthy individuals can modulate learning and memory. The purpose of this brief review was to evaluate how tDCS can be used to modulate implicit motor sequence learning and consolidation with the SRTT. So far, only six studies have addressed this question and most studies have tackled frontal brain areas.

For M1, bilateral anodal stimulation can enhance implicit motor sequence *learning* and probably also *consolidation* (Nitsche et al., 2003b; Kang and Paik, 2011; Kantak et al., 2012). This result is in line with previous studies which showed that M1 neurons are more responsive to tDCS than other cortical areas due to their morphology (Radman et al., 2009). Regarding consolidation, the results are not that clear yet and thus, further research is necessary to investigate the role of M1 for both GMS and SS consolidation. Nevertheless, as in both the studies by Nitsche et al. (2003b) and by Kang and Paik (2011), performance in the anodal or cathodal stimulation group was compared to the sham group separately rather than in a full ANOVA, the effects may have been overestimated. Importantly, future studies should also take SRTT parameters into account. Neurophysiological data have shown that the application of tDCS during an intense motor practice phase can impair motor performance while less intense practice can improve performance (Bortoletto et al., 2015). This suggests that the behavioral effects of tDCS are the result of an interaction between excitability changes induced by tDCS and by practice (Miniussi et al., 2013). Hence, the quantity of practice during the SRTT could influence tDCS effects.

For PM, there is not much evidence that tDCS might modulate implicit motor sequence learning (Nitsche et al., 2010; Kantak et al., 2012). If present, the effects seem to appear only immediately after tDCS (Nitsche et al., 2010; Kantak et al., 2012). Future studies should systematically vary tDCS parameters such as electrode size and shape, current length, and strength. This may be a promising avenue as neuroimaging studies have shown the involvement of PM in implicit motor sequence learning (Peigneux et al., 2000).

For PFC, only one study was available and this study did not find any modulating effects (Nitsche et al., 2003b). However, it is possible that more difficult sequence learning paradigms may be modulated by PFC stimulation. For example, there is evidence for the critical role of PFC in task sequence learning (Meier et al., 2013) Moreover, a recent study found that tDCS applied above the right PFC modulated performance in a probabilistic sequence learning task in which only every second element was sequenced (Janacsek et al., 2015).

For the cerebellum, there is initial evidence that off-line tDCS can enhance both GMS and SS consolidation (Ferrucci et al., 2013). This is in line with the hypothesis that the cerebellum is more responsive to tDCS compared to cerebral cortex areas (Rampersad et al., 2014).

So far, no study has evaluated the influence of supplementary motor area tDCS on implicit motor sequence learning and consolidation. This area can be easily tackled with tDCS and findings from neuroimaging and neurostimulation studies suggest its critical involvement in implicit motor sequence learning (Hazeltine et al., 1997; Kim and Shin, 2014). Therefore, future studies should also address the effect of supplementary motor area tDCS. Similarly, no study has evaluated the effects of parietal tDCS for implicit motor sequence learning and consolidation. Previous studies have shown that parietal cortex tDCS can influence memory encoding (Jacobson et al., 2012). Moreover, parietal activation has been found in neuroimaging studies of motor learning and motor learning consolidation (Doyon et al., 2009; Albouy et al., 2015). In addition, because parietal tDCS may activate cortico-hippocampal networks, it could help to disentangle the role of these networks (Reber, 2013; Wang et al., 2014; Dudai et al., 2015). This may motivate future studies with parietal tDCS.

## Conclusions

So far the most robust evidence for a modulating effect of tDCS on implicit motor sequence learning concerns the primary motor cortex (M1). Different studies have found that tDCS delivered on-line can enhance performance. There is also initial evidence for the modulating effect of off-line tDCS to the cerebellum. Evidence for PM stimulation is not robust, while evidence for PFC stimulation is negative. Further studies are required to address the effect of stimulation on different brain regions, different task parameters (e.g., number of sessions, see Meinzer et al., 2014), and different tDCS parameters (e.g., current intensity, see Cuypers et al., 2013). In any case, the investigation of the possibilities to modulate learning and consolidation with tDCS is still in its infancies and a more systematic examination of both task properties and stimulation parameters is warranted.

## Author contributions

All authors listed, have made substantial, direct and intellectual contribution to the work, and approved it for publication.

### Conflict of interest statement

The authors declare that the research was conducted in the absence of any commercial or financial relationships that could be construed as a potential conflict of interest.

## Footnotes

^1^When GMS learning is assessed as the speed-up of RTs across sequenced blocks it may also involve some implicit sequence learning. In contrast, when it is assessed as speed-up of RTs across random blocks it can be considered as pure measure.
